# Proof of concept for the simplified breakdown of cellulose by combining *Pseudomonasputida* strains with surface displayed thermophilic endocellulase, exocellulase and β-glucosidase

**DOI:** 10.1186/s12934-016-0505-8

**Published:** 2016-06-10

**Authors:** Iasson E. P. Tozakidis, Tatjana Brossette, Florian Lenz, Ruth M. Maas, Joachim Jose

**Affiliations:** Institute of Pharmaceutical and Medicinal Chemistry, Westfälische Wilhelms-Universität Münster, PharmaCampus, Corrensstraße 48, 48149 Münster, Germany; NRW Graduate School of Chemistry, Westfälische Wilhelms-Universität Münster, PharmaCampus, Corrensstraße 48, 48149 Münster, Germany; Autodisplay Biotech GmbH, Merowingerplatz 1a, 40225 Düsseldorf, Germany

**Keywords:** Whole cell biocatalysis, Cellulose breakdown, Glucose production, *Pseudomonas putida*, Cellulases, Surface display, Autotransporter, Maximized autotransporter mediated expression (MATE)

## Abstract

**Background:**

The production and employment of cellulases still represents an economic bottleneck in the conversion of lignocellulosic biomass to biofuels and other biocommodities. This process could be simplified by displaying the necessary enzymes on a microbial cell surface. Such an approach, however, requires an appropriate host organism which on the one hand can withstand the rough environment coming along with lignocellulose hydrolysis, and on the other hand does not consume the generated glucose so that it remains available for subsequent fermentation steps.

**Results:**

The robust soil bacterium *Pseudomonas putida* showed a strongly reduced uptake of glucose above a temperature of 50 °C, while remaining structurally intact hence recyclable, which makes it suitable for cellulose hydrolysis at elevated temperatures. Consequently, three complementary, thermophilic cellulases from *Ruminiclostridium thermocellum* were displayed on the surface of the bacterium. All three enzymes retained their activity on the cell surface. A mixture of three strains displaying each one of these enzymes was able to synergistically hydrolyze filter paper at 55 °C, producing 20 μg glucose per mL cell suspension in 24 h.

**Conclusion:**

We could establish *Pseudomonas putida* as host for the surface display of cellulases, and provided proof-of-concept for a fast and simple cellulose breakdown process at elevated temperatures. This study opens up new perspectives for the application of *P. putida* in the production of biofuels and other biotechnological products.

**Electronic supplementary material:**

The online version of this article (doi:10.1186/s12934-016-0505-8) contains supplementary material, which is available to authorized users.

## Background

Lignocellulosic biomass represents a sustainable feedstock for the biotechnological production of both biofuels and various platform chemicals since it is abundantly available and can be utilized without adverse effects on the global food supply. It comprises up to 50 % cellulose, a linear homopolymer consisting of long chains of β-1,4-linked glucose units that are symmetrically assembled to a stable, partly crystalline structure [1]. This structure is embedded into a matrix of the heterogeneous polymers lignin and hemicellulose, making a physical and/or chemical pretreatment of the raw material necessary to get access to its cellulose content [2]. For its conversion into value-added products, cellulose needs to be depolymerized, which can be attained by the combined application of three types of cellulose-degrading enzymes (cellulases): Endocellulases (EC 3.2.1.4) randomly cleave internal glycosidic bonds, primarily at the amorphous regions of the polymer. The thereby generated chain ends are processed by exocellulases (EC 3.2.1.91), yielding mainly disaccharide units. Finally, β-glucosidases (EC 3.2.1.21) hydrolyze the remaining glycosidic bonds to form glucose, which can then—simultaneously or in a separate step—be converted to the desired product by a fermenting microorganism [3].

By now, the wide commercialization of such a process has significantly been hampered by the lack of appropriate expression platforms that allow a cost-effective production and employment of cellulases. Currently, industry uses enzyme mixtures produced by the cellulolytic fungus *Trichoderma reesei*, which despite high enzyme yields is still considered as too costly to be used for economic large-scale applications [4]. Therefore, different strategies for the recombinant expression of cellulases have been developed [5]. Among those, the attachment of the enzymes to the exterior of a microbe, a technique termed surface display, has been identified as a promising approach that could reduce the high operational costs linked to cellulose degradation by limiting the necessary process steps to a minimum [6]: Microbial cells expressing surface displayed cellulases can directly be applied to the pretreated raw material; in contrast to intracellularly expressed enzymes, cell disruption and enzyme purification steps can be omitted. Since the expressed enzymes are connected to the microbial cells, they can be reused by harvesting the whole cells from the reaction mixture [7]. However, it is not easy to express satisfactory amounts of the at least three necessary types of enzymes in a single microbial cell. In contrast, their separated expression keeps the metabolic burden on the microbial cells low. Furthermore, cellulases can exert a high degree of synergism, meaning the cellulolytic activity of the combined enzymes can reach higher levels than the sum of the individual cellulase activities [8]. Several studies showed that the degree of synergism strongly depends on the ratio of the applied cellulases, which implies that precise control over the composition of the enzyme mixture is important to maximize hydrolysis efficiency [9–11]. Such a control is difficult to obtain when a single organism produces multiple enzymes. Additionally, different types of lignocellulosic materials require different enzyme mixtures; this applies not only for cellulases in the narrow sense, but also for accessory enzymes that promote or facilitate the hydrolysis reaction, e.g. hemicellulases, xylanases, and polysaccharide monooxygenases [12, 13]. The availability of a set of bacterial strains that can be cultivated easily and in large amounts, each displaying a single enzyme, would bring a great deal of flexibility and simplicity into the lignocellulose degradation process.

**Fig. 1:**
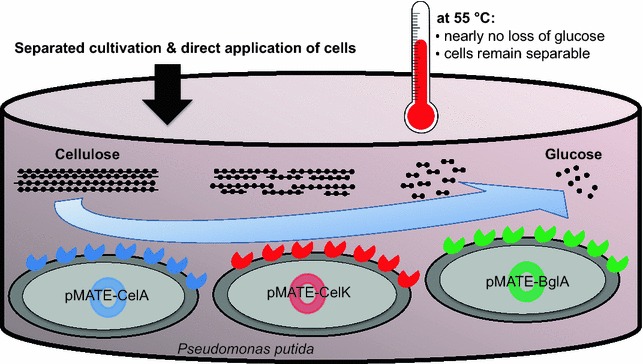
The herein described approach for a low-cost degradation of cellulose using a mixture of three *P. putida* strains displaying endocellulase CelA, exocellulase CelK and β-glucosidase BglA, respectively

Due to its advanced establishment in industry, yeast has been reported most often as host species for the display of cellulases [14–18]. When it comes to minimizing the complexity and hence the costs of a process, bacteria offer certain benefits such as higher growth rates, higher protein production and reduced cultivation demands. Still, surface display of cellulases on bacteria has to date almost exclusively been approached with *Bacillus subtilis* and *Escherichia coli* as hosts [19–26]. However, to ensure the necessary stability and reusability of a whole cell catalyst, a host is required that tolerates the harsh cultivation and reaction conditions coming along with industrial scale cellulose hydrolysis. In this regard, the soil bacterium *Pseudomonas putida* appears well suited, as it can withstand adverse factors such as extreme temperature and pH, various toxins and organic solvents [27]. Its potential industrial applications range from the production of polyhydroxyalkanoates [28] over the synthesis of natural products such as rhamnolipids, terpenoids or amino acid derivatives [29] to the recombinant expression of proteins [30]. In the latter application, *Pseudomonas* strains have already gained commercial relevance as an alternative to *E. coli* [31]. So far, *P. putida* has not been used as a platform for the degradation of lignocellulosic biomass. We have recently developed a surface display system named MATE (maximized autotransporter mediated expression), which is based on the autotransporter secretion mechanism using EhaA from *E. coli*, and which was specifically designed for its application in a broad range of Gram-negative bacteria [32]. In this study, MATE was used to display three complementary, thermophilic cellulases from *Ruminiclostridium thermocellum* on the surface of *P. putida* KT2440. It could be demonstrated that all three enzymes retained their hydrolytic activity on the bacterial cell surface. With these whole cell catalysts at hand, we assessed the principal feasibility of combining them for a fast and simple degradation of cellulosic substrates (Fig. 1). It turned out that a mixture of the three strains was able to synergistically degrade filter paper at 55 °C. Importantly, at this temperature the employed *P. putida* cells remained structurally intact, but did not take up the generated glucose, which opens up the possibility to recover the enzyme-bearing cells and use the reaction medium in a subsequent fermentation process.

## Results

### Glucose uptake and recyclability of *P. putida* at different temperatures

**Fig. 2:**
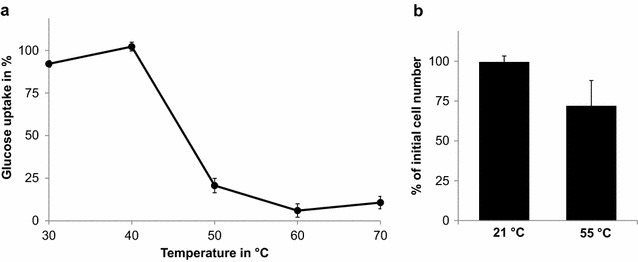
**a** Glucose uptake by *P. putida* at temperatures between 30 and 70 °C. The cells were resuspended in 50 mM sodium citrate buffer, pH 6, to an optical density of 20 and incubated with 1 mg/mL glucose in 50 mM sodium citrate buffer for 20 h. The glucose concentration in the cell supernatant was monitored by means of a DNS assay. **b** Recovery rates of *P. putida* cells after being incubated for 24 h at 21 and 55 °C. Post incubation the cell suspensions were centrifuged and the separated cells quantified by means of flow cytometry

For the economic success of a cellulose hydrolysis process it is crucial that the generated glucose can be recovered from the reaction mixture to be available for a subsequent fermentation step. This is unproblematic when using purified cellulases. However, when employing whole cells as catalysts, glucose might be consumed in the course of the hydrolysis reaction as it constitutes a convenient carbon source for most microbes. We therefore assessed the glucose-uptake of *P. putida* by incubating the bacterium with 1 mg/mL glucose and at temperatures from 30 to 70 °C. After 20 h, the glucose concentration in the cell supernatant was measured by means of a 3,5-dinitrosalicylic acid (DNS) assay [33] (Fig. 2a), and found that at temperatures of 50 °C and above *P. putida* did take up only approx 20 % of the glucose, making it feasible to run a cellulose hydrolysis process at this temperature without losing too much glucose to the host’s metabolism. To assess if *P. putida* remained structurally intact and hence recycable under these reaction conditions, the cells were incubated at 55 °C for 24 h and subsequently harvested by centrifugation. Before and after incubation, the cell number was determined by means of a flow cytometer. *P. putida* cells that were incubated at room temperature (21 °C) for the same period were used as a control. While at 21 °C nearly 100 % of the cells could be recovered, the cell number decreased to about 70 % when incubated at 55 °C. Nevertheless, this is still a high degree of recovery and thus indicates the suitability of *P. putida* as a host for the construction of recombinant whole cell catalysts supposed to work at elevated temperatures (Fig. 2b).

### Construction of MATE-cellulase fusion genes

**Fig. 3:**
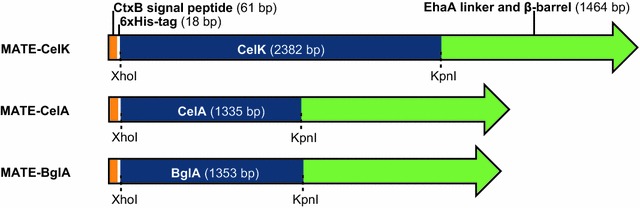
Schematic structure of the constructed MATE fusion genes as encoded by the used pMATE vectors. Cellulase genes CelK, CelA and BglA were relieved from unnecessary domains, synthesized commercially with flanking *Xho*I and *Kpn*I restriction sites (indicated by *dashed lines*), and ligated into pMATE expression vectors [32]. Domain lengths in base pairs (bp) are denoted in brackets

Based on these results, the previously described maximized autotransporter mediated expression (MATE) [34] was exploited to display the β-glucosidase BglA [GenBank: X60268], the exocellulase CelK [GenBank: AF039030] and the endocellulase CelA [GenBank: K03088], all from the thermophilic bacterium *Ruminiclostridium thermocellum,* on the surface of *P. putida.* To this end, expression vectors were constructed that encode MATE fusion proteins (named MATE-BglA, -CelK and -CelA, respectively) consisting of three essential parts, namely (from N- to C-terminus) (1) the signal peptide of the *Vibrio cholerae* toxin B subunit (CtxB), which is necessary for the translocation of the protein into the periplasm by the Sec pathway and has been proven useful for surface display applications [35]; (2) the respective enzyme domains BglA (encoded by plasmid pMATE-BglA), CelK (pMATE-CelK) and CelA (pMATE-CelA) and (3) a modified version of the EhaA AT translocation unit [32], which contains a linker and a β-barrel region responsible for incorporation into the outer membrane and display of the cellulase on the cell surface [36–38]. In between signal peptide and cellulase domain, a 6xHis epitope was included to allow an immunological verification of the cellulases surface localization by flow cytometry (Fig. 3). The expression of these fusion proteins was subjected to the control of an inducible araBAD promoter.

### Expression and localization of MATE-cellulase fusion proteins

**Fig. 4:**
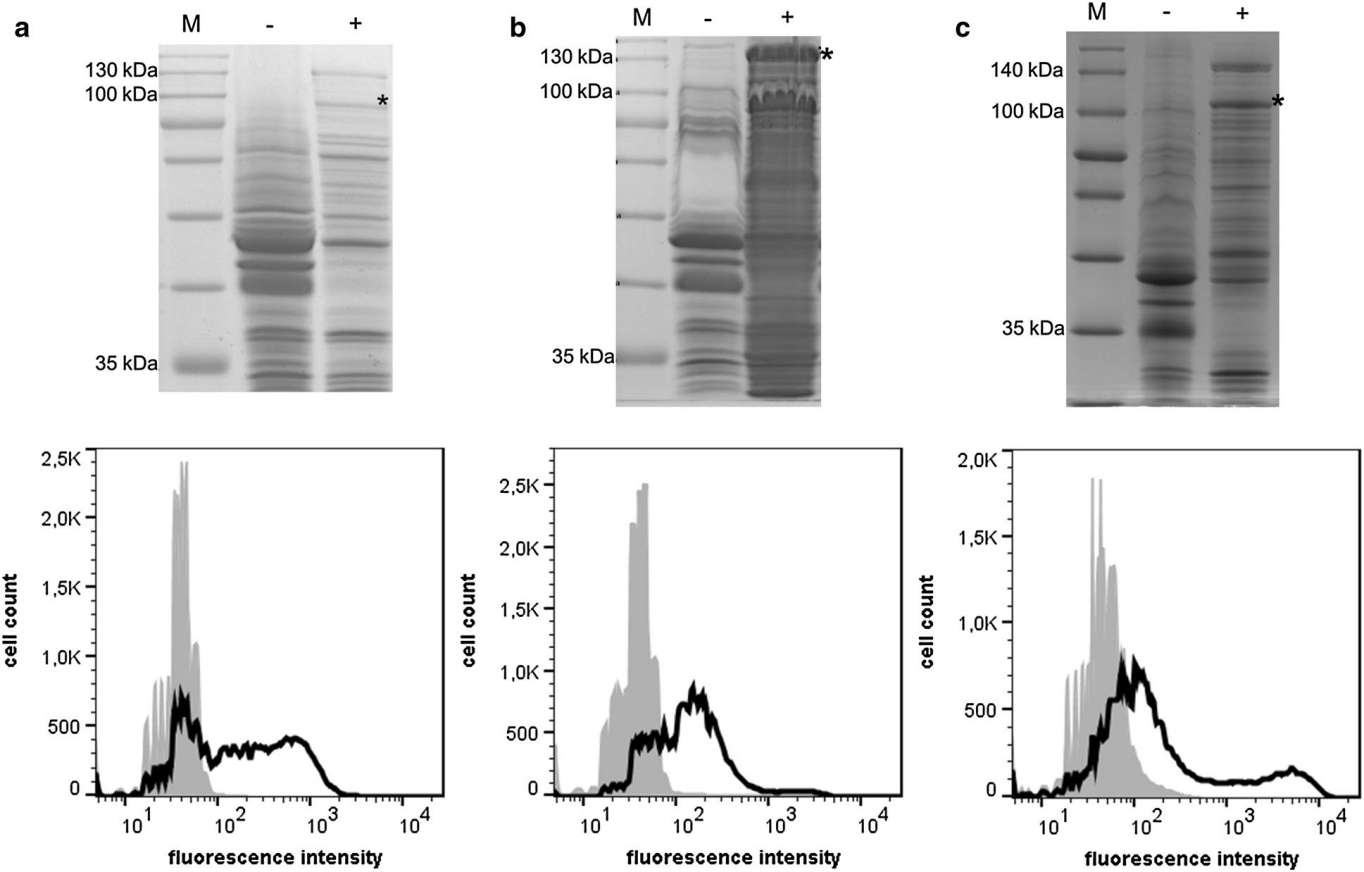
*Above* Coomassie-stained SDS-PAGE of outer membrane proteins of cells expressing MATE-BglA **(a)**, MATE-CelK **(b)** and MATE-CelA **(c)**. Samples of cells with (+) and without (−) recombinant protein expression are shown. The sizes of relevant marker proteins (*M*) are denoted. The *asterisks* (*) indicate the bands assigned to the MATE fusion proteins. *Below* Flow cytometer analysis of *P. putida* cells expressing MATE-BglA (**a**), MATE-CelK (**b**) and MATE-CelA (**c**). *Black* *P. putida* cells expressing the respective MATE fusion protein. *Grey* Control cells without protein expression. The cells were cultivated to an OD of 0.5, protein expression was induced by the addition of 0.2 % l-arabinose, and after 4 h of further cultivation the culture was centrifuged. For SDS-PAGE analysis, outer membrane proteins of the respective cells were isolated according to a modified protocol of Park et al. [57]. For flow cytometer analysis, the cells were resuspended in PBS to a final OD of 0.4. After washing three times with PBS, the cells were incubated with a 1:50 solution of mouse anti-6xHis antibody for 30 min at RT, washed three times and then incubated with a 1:100 DyLight-633-coupled anti-mouse antibody for one hour at RT. After washing three times, the fluorescence of 50,000 individual cells was analyzed

The plasmids pMATE-BglA, pMATE-CelK and pMATE-CelA were inserted into *P. putida* KT2440 by electroporation. The cells did not show any growth slowdown or defect when maintaining these plasmids. The induction of protein expression did not alter the viability of *P. putida* (Additional file 1: Figure S1). To confirm the expression of the encoded fusion proteins as well as their localization within the bacterial outer membrane, the cells were cultivated, protein expression was induced by the addition of l-arabinose, and outer membrane proteins of each strain were isolated and analyzed by SDS-PAGE (Fig. 4, upper panel). *P. putida* cells without recombinant protein expression were used as controls. Coomassie staining of the separated outer membrane proteins revealed the presence of a weak protein band at an apparent molecular weight of approx. 100 kDa in samples of *P. putida* pMATE-BglA (Fig. 4a, above). As this protein was not visible in samples of the control cells, it was assigned to MATE-BglA, which has a predicted molecular weight of 109 kDa. In the case of *P. putida* cells carrying pMATE-CelK, outer membrane protein samples showed a strong band at the expected molecular weight of MATE-CelK (147 kDa), which did not appear in the control sample (Fig. 4b, above). Samples of cells expressing MATE-CelA showed a protein band at approx. 100 kDa, which was not visible in the respective control sample and thus appeared to be the MATE-CelA fusion protein with a predicted molecular weight of 106 kDa (Fig. 4c, above). These experiments indicated that the constructed MATE-vectors were able to direct the expression of the desired fusion proteins in *P. putida*, and that the N-terminal CtxB signal peptide from *V. cholerae* was properly recognized by this species, leading to the translocation of the proteins to the outer membrane.

### Surface display of cellulases on *P. putida*

To verify the actual display of the enzymes on the cell surface, a flow cytometer analysis was performed (Fig. 4). To this end, *P. putida* cells expressing MATE-CelK, -BglA and -CelA, respectively, were first treated with anti-6xHis antibody, followed by treatment with a DyLight-633-coupled second antibody. *P. putida* cells without recombinant protein expression were used as control. Due to their size, antibodies are not able to cross the bacterial outer membrane and therefore can only bind to an epitope when it is accessible at the surface of the cells. As can be seen in the flow cytometry histogram, all three *P. putida* strains expressing the fusion proteins exhibited a significantly higher mean fluorescence [MATE-BglA: mean fluorescence (mF) = 370, MATE-CelK: mF = 275, MATE-CelA: mF = 868] in comparison to cells without protein expression (mF = 60–82). This represented strong evidence for the localization of the cellulases on the outside of the cells.

### Activity of MATE-cellulases on the surface of *P. putida*

**Fig. 5:**
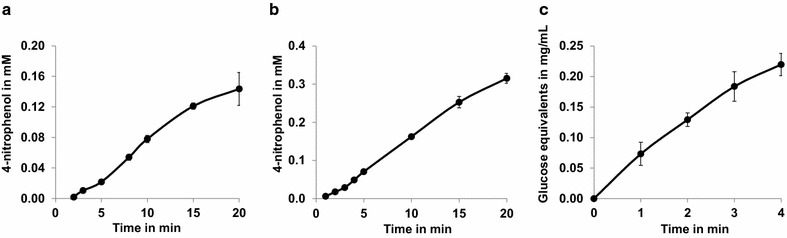
Whole cell activity assays of *P. putida* displaying BglA (**a**), CelK (**b**) and CelA (**c**). The cells were cultivated to an OD of 0.5, protein expression was induced by the addition of 0.2 % l-arabinose, and after 4 h of further cultivation the cells were harvested and resuspended in 50 mM sodium citrate buffer, pH 6, to a final OD of 1 (CelK) and 40 (BglA and CelA). The cells were then mixed 1:1 with 10 mM 4-nitrophenyl-β-d-glucopyranoside (BglA), 10 mM 4-nitrophenyl-β-d-cellobioside (CelK) or 2 % carboxymethylcellulose (CelA) and incubated at 55 °C. For BglA and CelK, the generated 4-nitrophenol was quantified by measuring the absorption of the cell supernatant at 405 nm. For CelA, the amount of reducing sugars in the cell supernatant was determined by means of a DNS assay [33]. *P. putida* without plasmid served as control, no hydrolytic activity could be detected with these cells (not shown)

To find out whether the cellulases retained their activity on the surface of *P. putida*, photometric whole cell activity assays were performed at 55 °C. BglA-displaying cells were incubated with 5 mM 4-nitrophenyl-β-d-glucopyranoside and CelK-displaying cells with 5 mM 4-nitrophenyl-β-d-cellobioside. As can be seen in Fig. 5a, b, both strains exhibited significant hydrolytic activity towards their substrates as judged by a continuously increasing absorption value at 405 nm caused by the formation of 4-nitrophenol. The linear ranges of the absorption increases (5–15 min for BglA- and 4–15 min for CelK-displaying cells) was used to calculate activities of 0.5 mU/mL_OD1_ for BglA- and 37.1 mU/mL_OD1_ for CelK-displaying cells. The enzymatic activity of CelA-displaying cells was determined with carboxymethylcellulose (CMC) as substrate. For this purpose, the cells were incubated in a 1 % CMC solution, and the cell supernatant was subjected to a DNS assay [33], giving a color reaction that is proportional to the amount of reducing sugars and can be quantified photometrically at 540 nm. As shown in Fig. 5c, CelA-displaying *P. putida* cells catalyzed the formation of reducing sugars from CMC. Based on the linear range of 1–3 min, an activity of 15.3 mU/mL_OD1_ was calculated.

### Recyclability of cellulase-displaying *P. putida* strains

**Fig. 6:**
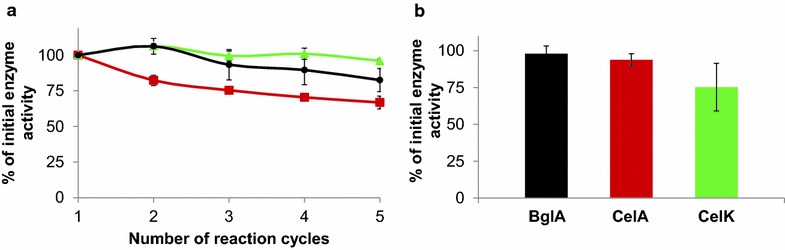
**a** Enzyme activity of *P. putida* cells displaying BglA (*black*), CelA (*red*) and CelK (*green*) after repeated reaction cycles. Activity assays were performed as described in Fig. 5. After each assay, the cells were harvested by centrifugation and resuspended in fresh substrate solution. The obtained activity data from time points within the linear ranges of substrate conversion (7 min for BglA-, 10 min for CelK-, 2 min for CelA-displaying cells) were normalized to the activity of the cells in the first reaction cycle. **b** Activity of the cells after being preincubated at 55 °C for 24 h

One of the major advantages of using whole microbial cells for catalytic applications is their reusability in multiple reaction cycles. To find out if this is true for the generated cellulase-displaying *P. putida* strains, the described activity assays were performed in a consecutive manner, i.e. after each activity assay the cells were harvested by centrifugation and resuspended in a fresh substrate solution for another reaction cycle. This procedure was repeated 5 times as it has been done previously for whole cell biocatalysts displaying nitrilase [39] and prenyltransferase [40]. As can be seen in Fig. 6a, BglA-displaying cells exerted a residual β-glucosidase activity of approx. 82 % after five repeated applications. CelA-displaying cells had 67 % of their original endocellulase-activity and CelK-displaying cells retained 96 % of their exocellulase-activity. Even beyond this number of reactions the cells were still reusable, with residual activites of 71 % after 9 cycles (BglA), 80 % (CelK) and 51 % (CelA) after 10 cycles (data not shown).

To assess the thermal stability of the surface-displayed cellulases, the described activity assays were done with each of the three strains after being incubated at 55 °C for 24 h (Fig. 6b). In case of BglA- and CelA-displaying cells nearly no loss of enzymatic activity could be detected. CelK-displaying cells reduced their activity to about 75 %.

### Concerted hydrolysis of filter paper

**Fig. 7:**
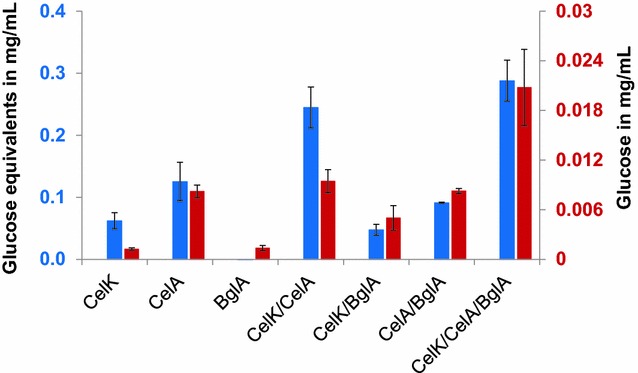
Degradation of filter paper by cellulase-displaying *P. putida* cells and mixtures thereof. The cells were cultivated to an OD of 0.5, protein expression was induced by the addition of 0.2 % l-arabinose, and after 4 h of further cultivation the cells were harvested and resuspended in 50 mM sodium citrate buffer, pH 6, to a final OD of 16.6. 23.6 µL of each cell suspension was added to the mixtures as indicated, filled up with buffer when necessary to a total volume of 71 µL, and incubated for 24 h with a round patch (6 mm diameter) of Whatman filter paper No. 1 at 55 °C. Subsequently, the cells were removed, reducing sugars were quantified via DNS assay (*blue*) and glucose was specifically determined by means of a commercial reagent kit (*red*)

The filter paper assay [41] is the most commonly used assay to determine total cellulase activity. We therefore used it to investigate the hydrolytic performance of a mixture of the generated *P. putida* strains. For this purpose, each strain as well as different combinations of two and three strains were applied for 24 h to filter paper according to a protocol of Xiao et al. [42], followed by both a DNS assay to detect the released reducing sugars and a specific glucose detection assay (Fig. 7).

The application of CelK-displaying cells alone lead to an increased amount of reducing sugars due to the release of cellobiose as a product of exocellulase-activity. As expected, no glucose was formed by single reaction with CelK. A considerable amount of reducing sugars could also be detected when applying CelA-displaying cells alone, which is consistent with the fact that filter paper contains amorphous (non-crystalline) regions that can be processed by the endocellulase, generating new chain ends which are detected by the DNS assay. Surprisingly, incubation of filter paper with these cells led to a relatively large amount of glucose although actually an is endocellulase is expected to release only longer cellulose fragments. According to these observations, a combination of CelA- with CelK-displaying cell yielded a similar quantity of glucose as with CelA-displaying cells alone, but more than twice the amount of reducing sugars compared to samples with CelA- or CelK-displaying cells alone. This could reflect an increase of available open chain ends for CelK due to the activity of CelA, allowing a synergistic activity of these two cellulases. BglA-displaying cells alone did not show a considerable effect on the filter paper. This is plausible as the displayed β-glucosidase can solely degrade cellobiose, which is not present without preceding exocellulase-activity. As expected, the addition of BglA- to CelA-displaying cells also did not have an effect, since CelA activity cannot produce any substrate for BglA. Combining CelK- with BglA-displaying cells resulted in a significant increase of glucose in the reaction medium, however no increase of reducing sugars could be detected by means of the DNS assay. A similar observation was made when adding BglA-displaying cells to a mixture of CelK- and CelA-displaying cells: Compared to the CelK/CelA-mixture, only a slight increase of reducing sugars was detectable, but the specific glucose assay showed that the amount of glucose was more than double as high in samples of CelK-, CelA- and BglA-displaying cell mixtures. The combination of all three strains yielded 20 μg glucose per mL cell suspension. Taken together, these experiments prove that a mixture of the generated *P. putida* strains was able to synergistically hydrolyze filter paper and identified the β-glucosidase as the current bottleneck.

## Discussion

Displaying cellulases on the surface of microbial cells offers some advantages that could make the degradation of lignocellulose more cost-efficient. Therefore several groups have studied various approaches, which basically differ in the used host organism, surface display method, type and origin of the displayed cellulases, and connected to that, their expression as a separate enzyme or as a complexed part of a cellulosome [6]. The host organism used for expressing and displaying the desired enzymes determines important characteristics of the generated whole cell catalyst such as growth rate, enzyme activity and stability. The availability of an appropriate host organism thus is crucial for the transfer of the cellulase display concept to industrial applications. Yeast, due to its advanced establishment in industry, has been reported most often as host species for cellulase display applications. Examples for the display of multiple individual cellulases [17, 43] as well as the display of cellulosomes [16, 18] have been reported. Surface display of cellulases on bacteria has to date mostly been approached with the Gram-positive *Bacillus subtilis* and the Gram-negative *E. coli* as hosts. *B. subtilis* has especially been used for the display of cellulosomes [19, 26]. In contrast, *E. coli* has so far only been reported as host for the display of one or more non-complexed cellulases using various outer membrane anchoring motifs [20, 21, 24, 25], among them also the AIDA-I autotransporter [23]. The only exception from using these established model organisms was reported by Kojima et al., who displayed an endocellulase on the surface of the ethanologenic bacterium *Zymobacter palmae* using the ice nucleation protein anchoring motif from *Pseudomonas syringae* [44]. Because of its versatile metabolism, ease of genetic manipulation and resistance towards various adverse conditions, *P. putida* has in recent years strongly developed towards industrial applications [27]. However, it has never before been used as a platform for surface-displayed cellulases. In this study, we therefore aimed to create a simple and flexible cellulolytic system based on this bacterium.

For a cellulose hydrolysis process in which the generated glucose is not directly converted to the desired product, but instead is intended to be utilized in a separate fermentation step, it is of primary interest that the sugar can be recovered in high yields from the hydrolysate. For a whole cell catalytic approach as presented here, this means that the employed host cells must not take up and metabolize glucose at the chosen reaction conditions. We found that at 50 °C and above nearly no glucose was consumed by *P. putida*, presumably because its glucose transporters and/or metabolic enzymes were denatured. After 1 day of incubation at a temperature of 55 °C, the largest part of the cells was still separable from the reaction mixture by centrifugation. This is particularly important for industrial scale applications, in which a recovery of the cells for repeated hydrolysis cycles is desirable, and emphasizes one of the advantages of using *P. putida* for the described process.

The MATE system used for displaying the cellulases is based on the EhaA autotransporter from *E. coli*, which has previously been shown to be applicable for surface display in *E. coli* itself [32, 45] and also in the ethanologenic bacteria *Z. palmae* and *Zymomonas mobilis* [46]. In this study, the expressed MATE fusion proteins could be found in the outer membrane of *P. putida*, providing evidence that their N-terminal CtxB signal peptide was properly recognized by the organism’s Sec machinery and consequently triggered their translocation into the periplasmic space. Flow cytometry measurements showed that all three cellulases were successfully exposed to the extracellular space of *P. putida*, which proves that the EhaA transporter domains are functional in this species and allow the display of recombinant proteins on its cell surface. Previously reported incompatibilities between AT and host organism [47, 48] and the disability of an AT to display heterologous proteins [49] therefore seem not to be a problem in this case, and suggest that MATE can be used in a broad range of host bacteria.

Whole cell activity assays showed that all three enzymes retained their functionality on the cell surfaces and exhibited hydrolytic activity towards their substrates. The cells could be recycled for consecutive reactions and retained between 67 and 96 % of their initial activity after five cycles. These are very high residual activities when compared to previous studies, in which a prenyltransferase retained 23–46 % [40] of its activity after three repeated uses and a nitrilase 55 % after five reaction cycles [39]. The chosen reaction temperature of 55 °C reflects the consideration of the previously mentioned uptake of glucose and structural stability of *P. putida* as well as the enzymes temperature optima, which are reported to be 60 °C for BglA [50], 65 °C for CelK [51] and 75 °C for CelA [52]. According to these optima, we experienced higher catalytic activities of the whole cell catalysts when increasing the temperature. For example, the activity of *P. putida* cells with surface displayed CelA was more than double as high at 75 °C than at 55 °C (92.78 mU/mL_OD1_ compared to 41.89 mU/mL_OD1_, endpoint measurement; data not shown). Thus, from the viewpoint of enzyme activity a raise of temperature beyond 55 °C appears beneficial, but could impair the host cells structural integrity. To resolve this mismatch, one could apply enzymes with a lower temperature optimum, or establish a more thermostable bacterium as expression host for the MATE system. The first approach would require bacterial cellulases that are highly efficient at low temperatures; enzymes with such characteristics are currently not available. Following the second approach, it has to be taken into account that the cultivation of thermophilic bacteria and their use for protein expression is difficult and energy-intensive. This could collide with the intended simplicity and cost-efficiency of the process.

As enzymes represent the largest cost contributors to the conversion of cellulosic biomass to fuels or other chemicals, the commercial success of such a process depends on the ability to produce very large amounts of enzymes at reasonable costs [53]. When employing microbes instead of purified enzyme cocktails to achieve a cost reduction, it could be problematic to express satisfactory amounts of the at least three necessary types of enzymes in a single microbial cell. In contrast, a separated expression of the enzymes keeps the metabolic burden on the microbial cells low and, more importantly, makes it possible to adjust the quantity of each enzyme in the mixture. Such a strategy has been evaluated previously by Baek et al, who displayed cellulases on yeast cells and determined an optimal cell ratio for a maximized hydrolysis efficiency [54]. An approach like this has not been followed with bacteria so far. In this study, we found that a mixture of exocellulase CelK- and endocellulase CelA-displaying *P. putida* cells produced twice the amount of reducing sugars from filter paper than cells displaying only one of these cellulases. This result showed, in accordance with the study of Baek et al., that the enzymes do not necessarily have to be displayed on the same cell surface to obtain a synergistic activity. Unexpectedly, CelA-displaying cells alone produced a relatively large amount of glucose from the filter paper. It is thinkable that the endocellulase preferably hydrolyzed the very ends of the cellulose chains because they were better accessible for the enzyme, resulting in an increased release of glucose. Another explanation could be that CelA, beside its endocellulase activity, also possesses a β-glucosidase functionality, however no reports to this issue are available so far.

The addition of β-glucosidase BglA-displaying cells resulted in a significant increase of glucose in the reaction mixture, demonstrating that BglA also participated in the hydrolysis process. However, while the mixture of all three strains was able to produce 300 μg/mL glucose equivalents, the actual yield of glucose was only 20 μg/mL. Although a direct comparison of these two quantities cannot be drawn, their dimensions make obvious that the BglA-displaying cells represented the limiting factor in the complete hydrolysis of filter paper. This could be due to a poor expression level as evidenced by a comparably low amount of MATE-BglA found in the outer membrane of the expressing bacteria. However, it is also conceivable that the activity of BglA was impaired due to its expression as a MATE-fusion protein. According to our experience, the fusion of an enzyme with an AT does in most cases not interfere with its activity, still there are examples in which the fusion lead to a reduced enzyme activity [39, 55]. For verification of both hypotheses a comparison between the specific activities of free and displayed enzyme would be necessary, the latter requiring the number of enzymes on the bacterial cell surface for calculation. When using *E. coli* as host, this number can be determined approximately by performing an SDS-PAGE analysis of outer membrane protein isolates and comparing the intensity of the autotransporter fusion protein band with the intensity of the OmpA protein band, which is known to be present in a constant number in the outer membrane of *E. coli*. Since an analogous reference protein has not been established for *P. putida* yet, the quantification of displayed enzymes on the surface of this host, and hence a comparison between the activity of free and displayed enzyme, is currently not feasible.

β-Glucosidases represent a known bottleneck in cellulose hydrolysis processes [56]. To solve this, BglA either has to be substituted by an enzyme with higher activity, or the amount of BglA-displaying cells in the mixture has to be increased. For further development of the presented concept, we are planning to (1) optimize the expression of the MATE-cellulases, e.g. in terms of used plasmid backbone, promoter, culture conditions, in order to achieve a higher number of enzymes on the cell surfaces, and (2) to systematically vary the cell mixtures in their composition to find out an optimal ratio. Beyond that, the replacement of filter paper with an industrially relevant, lignocellulosic substrate would be desirable to have a more realistic valuation basis for the concept at hand.

## Conclusions

We provided proof-of-concept for the application of the industrially promising bacterium *P. putida* as host for the surface display of cellulases, and showed that it is possible to combine bacteria with different cellulases on their surface to achieve a synergistic hydrolysis of cellulosic substrates. The achieved activities still have a rather conceptional than industrially applicable value and require optimization. Nevertheless, the herein proposed approach can serve as a starting point for the creation of a fast, simple and modularly expandable cellulose degradation system.

## Methods

### Bacterial strains and culture conditions

Expression experiments were carried out in *Pseudomonas putida* strain KT2440. Cultures were grown in lysogeny broth (LB) medium containing 50 µg/mL kanamycine when necessary. Main cultures were inoculated with 1 % (v/v) of an overnight culture, and grown under shaking (200 rpm) at 30 °C until reaching an optical density (OD) of 0.5. For induction of protein expression, l-arabinose was added to a final concentration of 0.2 % (w/v), and the cells were cultured for further 4 h before being harvested.

### Cellulase genes and construction of MATE expression vectors

All three cellulase genes were synthesized commercially (Life Technologies, USA), and were exempted from domains which are not necessary for their hydrolytic activity or interfere with their surface translocation. For BglA [GenBank: X60268], this was only the start codon. In the case of CelA [GenBank: K03088], the 96 bp encoding for a signal peptide at the N-terminus were removed. CelK [GenBank: AF039030] was synthesized both without the 81 bp sequence encoding for an N-terminal signal peptide and the 234 bp sequence encoding for a C-terminal dockerin domain. Restriction sites for *Xho*I at the 5′ ends and *Kpn*I at the 3′ ends were added. Plasmids pMATE-CelK, pMATE-CelA and pMATE-BglA were constructed by inserting these genes via *Xho*I/*Kpn*I restriction into a vector described in a previous study [46], encoding for a MATE fusion protein as described in detail in another report [32], containing the replication origin and kanamycine resistance gene from pBBR1-MCS2, and *ara*BAD, *araC* and *rrn*B terminator from pBAD/gIII, with the difference of being devoid of a mob gene, as it is not necessary for replication in *P. putida*.

### Outer membrane protein analysis

Outer membrane proteins were prepared according to a modified protocol of Park et al. [57]. Shortly, the cell pellet was resuspended in 1.5 mL of 0.2 M Tris/HCl (pH 8.0) buffer after washing twice with the same buffer. 100 μL lysozyme (10 mg/mL in water), 100 μL saccharose (1 M in water), 100 μL EDTA (10 mM in water) and 3.2 mL water were added, followed by incubation for 15 min at RT. Subsequently, 10 μL aprotinin (10 mg/mL in 10 mM HEPES buffer pH 8.0), 50 μL phenylmethanesulfonylfluoride (100 mM in isopropanol), 5 mL extraction buffer (2 % Triton X-100, 10 mM MgCl_2_ in 50 mM Tris/HCl) and 100 μL DNAse (1 mg/mL in water) were added. After 45 min on ice, intact cells and cell debris were removed by centrifugation (5 min, 3200×*g*, 4 °C) and the supernatant was centrifuged for 10 min at 18,000 rpm and 4 °C. The resulting pellet was washed with 10 mL water, centrifuged again and resuspended in two volume equivalents SDS sample buffer (100 mM Tris/HCl [pH 6.8], 4 % SDS, 0.2 % bromophenol blue, 20 % glycerol). Proteins were separated by sodium dodecyl sulfate polyacrylamide gel electrophoresis (SDS-PAGE) using a 10 % polyacrylamide gel and made visible by staining with Coomassie Brilliant Blue.

### Flow cytometer analysis

Cell cultivation and protein expression was performed as described above. After harvesting the cells, they were washed twice with PBS, and incubated with mouse anti-6xHis antibody solution (1:50 in PBS, THE HisTag, GenScript, USA) for 30 min hour at RT, washed twice and subsequently incubated for 1 h with a DyLight-633 labeled second antibody solution (goat anti-mouse IgG, 1:50 in PBS, Thermo Scientific, USA) at RT. Cells were then washed twice and analyzed by means of a FACSAria III flow cytometer (BD Biosciences, USA) using an excitation wavelength of 633 nm.

### Exocellulase and β-glucosidase activity assay

To determine the hydrolytic activity of *P. putida* displaying CelK and BglA, respectively, cells were washed with 50 mM sodium citrate buffer pH 6.0 and resuspended in the same buffer to a final OD of 1 (CelK displaying cells) or 40 (BglA displaying cells), and preheated to 55 °C. 10 mM *p*-nitrophenyl-β-d-glucopyranoside (for BglA activity) respectively *p*-nitrophenyl-β-d-cellobioside (for CelK activity) solutions were also preheated to 55 °C and mixed 1:1 with the respective cells. After given timepoints, the cells were removed by centrifugation, and 100 μL of the supernatant was mixed with 100 μL of a 2 M Na_2_CO_3_ solution. The amount of released *p*-nitrophenol was detected photometrically at 405 nm in a plate reader.

### Endocellulase activity assay

To determine endocellulase activity, cells were prepared analogously to the previous section. OD was adjusted to 40, and the cells were mixed 1:1 with a preheated 2 % CMC solution (in 50 mM sodium citrate buffer pH 6.0). At given timepoints, cells were removed by centrifugation and the supernatant was subjected to a DNS assay as described below.

### Reusability experiments

For the determination of the cells residual enzymatic activity after consecutive reactions, the activities of the cells at a time point within the linear range of substrate conversion (7 min for BglA-, 10 min for CelK-, 2 min for CelA-displaying cells) were measured as described. Subsequently, the cells were harvested by centrifugation and resuspended in fresh substrate solution. The obtained values were normalized to the activities of the first reaction cycle.

### Filter paper assay

OD of all cell suspensions was adjusted to 16.6, and 23.6 µL of each cell suspension was added to the mixtures as indicated, filled up with buffer when necessary to a total volume of 71 µL, and a filter paper assay according to a protocol of Xiao et al. [42] was performed, with the following modifications: (1) The used filter paper discs had a diameter of 6 mm, resulting in an altered reaction volume of 71 μL. (2) The cells were incubated with the filter paper for 24 h at 55 °C. Subsequently, cells were removed and reducing sugars in the supernatant determined via DNS assay. (3) The reaction was performed in closed reaction tubes.

### Detection of reducing sugars and glucose

Reducing sugars were determined colorimetrically at 540 nm using the 3,5-dinitrosalicylic acid (DNS) assay, modified by King et al. [33], with the difference of using 50 μL reaction mixture with 100 μL DNS reagent. For the specific detection of glucose, the Glucose Detection Kit II (abcam, Cambridge, UK) was used.

## Additional file

10.1186/s12934-016-0505-8 Influence of MATE-fusion protein expression on the growth of *P. putida*. **Grey**: Cells without protein expression. **Black**: Cells expressing MATE-BglA (**A**), MATE-CelA (**B**) and MATE-CelK (**C**). Dashed lines depict the starting points of protein expression, which was induced by the addition of 0.2 % l-arabinose. Protein expression was conducted for 4 h. The solid lines depict the time points at which the culture was routinely harvested for further experiments.

## Abbreviations

CMC: caroboxymethylcellulose
DNS: 3,5-dinitrosalicylic acid
MATE: maximized autotransporter mediated expression
pNPG: 4-nitrophenyl-β-d-glucopyranoside
pNPC: 4-nitrophenyl-β-d-cellobioside
