# Supplementary figures and images for: Proof of concept for the simplified breakdown of cellulose by combining Pseudomonasputida strains with surface displayed thermophilic endocellulase, exocellulase and β-glucosidase
Source: Microb Cell Fact. 2016 Jun 10;15:103. doi: 10.1186/s12934-016-0505-8 (PMC4901517; doi:10.1186/s12934-016-0505-8)

## Slide 1
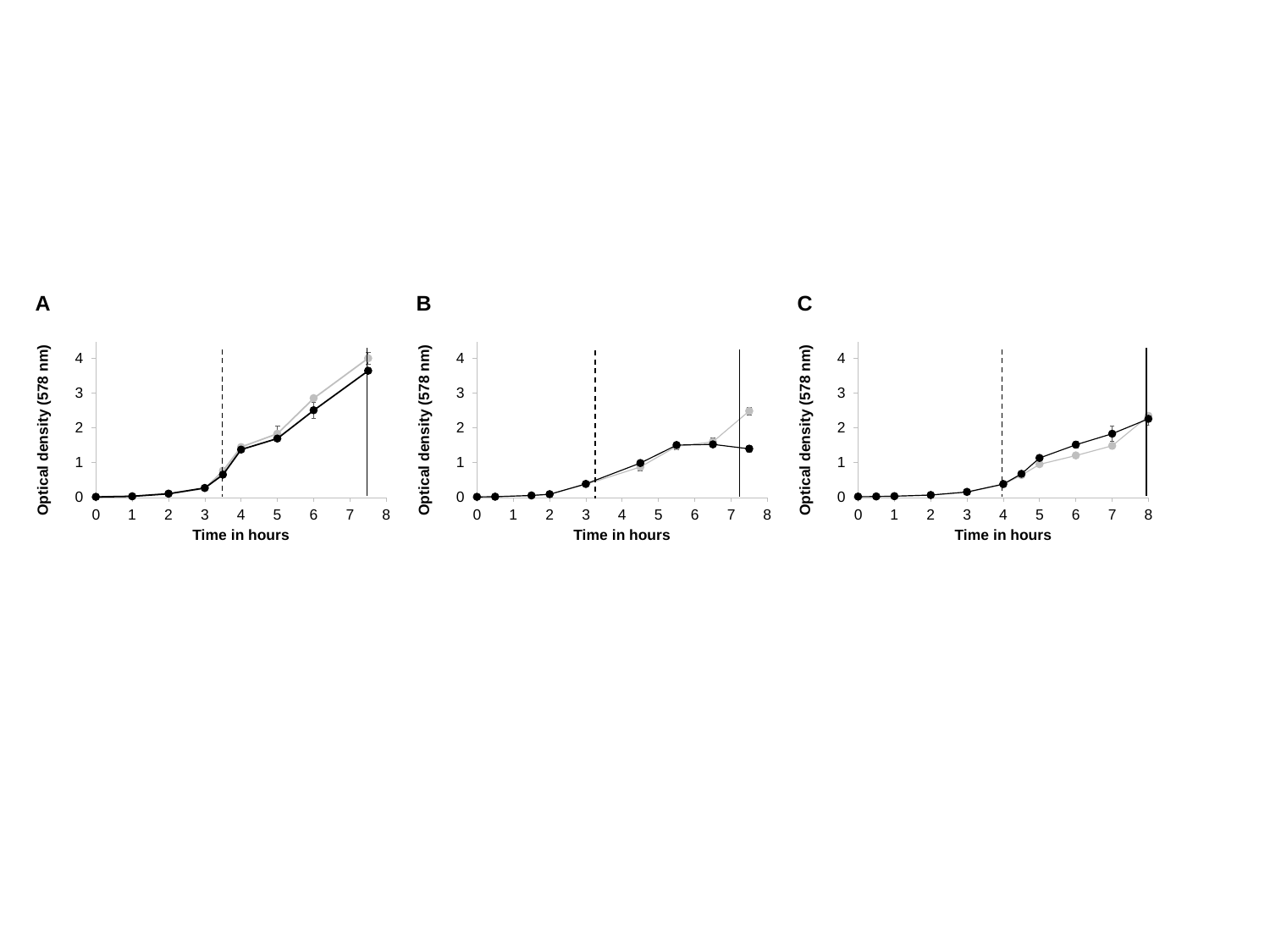

A
C
B

Supplement: Supplementary file 1 — 10.1186/s12934-016-0505-8 Influence of MATE-fusion protein expression on the growth of P. putida. Grey: Cells without protein expression. Black: Cells expressing MATE-BglA (A), MATE-CelA (B) and MATE-CelK (C). Dashed lines depict the starting points of protein expression, which was induced by the addition of 0.2 % l-arabinose. Protein expression was conducted for 4 h. The solid lines depict the time points at which the culture was routinely harvested for further experiments. [file 12934_2016_505_MOESM1_ESM.pptx]
